# Proteomics Integrated with Transcriptomics of Clubroot Resistant and Susceptible *Brassica napus* in Response to *Plasmodiophora brassicae* Infection

**DOI:** 10.3390/ijms26189157

**Published:** 2025-09-19

**Authors:** Kawalpreet Kaur, Dinesh Adhikary, Nat N. V. Kav, Sabine Scandola, R. Glen Uhrig, Habibur Rahman

**Affiliations:** 1 Department of Agricultural, Food and Nutritional Science, University of Alberta, Edmonton, AB T6G 2P5, Canada; kawalpre@ualberta.ca (K.K.); dadhika1@ualberta.ca (D.A.); nat.kav@ualberta.ca (N.N.V.K.); 2 Department of Biological Sciences, University of Alberta, Edmonton, AB T6G 2E9, Canada; scandola@ualberta.ca (S.S.); ruhrig@ualberta.ca (R.G.U.); 3 Department of Biochemistry, University of Alberta, Edmonton, AB T6G 2H7, Canada

**Keywords:** *B. napus*, clubroot, near-isogenic lines, ROS scavenging, cell wall modification, resistance

## Abstract

Clubroot disease, caused by *Plasmodiophora brassicae*, is a threat to *Brassica* crops; therefore, understanding of host-resistance is important for developing clubroot-resistant cultivars. Using multi-omics analysis of clubroot-resistant (CR) and -susceptible (CS) near-isogenic lines (NILs) of *B. napus*, carrying the resistance of turnip (*B. rapa* var. rapifera), we characterized the host resistance mechanisms. Through proteome analysis, we identified 6626 differentially abundant proteins (DAPs) (2353 in CR-NILs, 4273 in CS-NILs) (*q* < 0.05), of which 50 in CR- and 62 in CS-NILs were detected across the disease developmental stages. Notable proteins included those involved in reactive oxygen species scavenging (BnaA09T0647200WE)], cell-wall modifications (BnaA04T0244300WE) and glucosinolate biosynthesis (BnaA01T0266700WE) in the CR-NILs. Additionally, disease-resistance proteins like ENHANCED DISEASE RESISTANCE 2-like (BnaA03T0055600WE) and hairpin-induced family protein YLS9 (BnaA08T0237900WE) showed increased abundance in CR-NILs. In contrast, CS-NILs exhibited decreased abundance of defense-related proteins, including proteins containing CUPIN domain (BnaA09T0578800WE) and LACCASE (BnaA02T0019200WE). Integration of proteome data with transcriptome data revealed 33 genes in CR- and 32 in CS-NILs showing a consistent pattern, including the genes related to *PLANT INVERTASE/PECTIN METHYLESTERASE INHIBITOR* (BnaC04T0003100WE), *KELCH MOTIF* (BnaC02T0374800WE), *LACCASE* (BnaA02T0019200WE), and antioxidant-related transcripts [*GLUTATHIONE S-TRANSFERASES* (BnaA03T0280900WE) and *4-HYDROXYPHENYLPYRUVATE DIOXYGENASE* (BnaA09T0641500WE)]. Our findings offer valuable new targets for breeding clubroot-resistant *B. napus*.

## 1. Introduction

Canola (*Brassica napus* L.) is an important oilseed crop in the world. Its production is impacted by several biotic stresses including clubroot disease caused by a soil-borne obligate biotrophic protist *Plasmodiophora brassicae* Woronin [1,2]. This disease affects *Brassica* crops in more than 88 countries, resulting in 10–15% yield loss globally [3,4]. *P. brassicae* can survive in soil as resting spores for over 17 years. When the environmental conditions are favorable (20–24 °C temperature and wet soil with pH < 6.5), these spores germinate and cause primary infection on root hairs which is followed by secondary infection in root cortex. These events lead to an imbalance of phytohormones in the roots of infected plants, resulting in hypertrophy and hyperplasia of the roots, and eventually leading to the formation of characteristic club-shaped galls in the roots [5,6,7].

The agricultural practices, such as field equipment sanitation, soil amendments using lime, application of fungicides and boron, and crop rotation, have been proposed as management strategies of this disease [8]. However, they are not economical or effective in large cropping systems. To date, the deployment of clubroot-resistant cultivars has been the best practice; however, the emergence of new pathotypes capable of overcoming available host resistance poses a significant challenge [9]. This necessitates the identification of new resistance sources and understanding the genetic and molecular basis of the resistance for use in breeding to develop clubroot-resistant cultivars.

In recent years, studies using transcriptomics [10,11,12,13,14,15,16,17,18] and proteomics [19,20,21,22,23] approaches have been carried out to better characterize the molecular changes occurring during the interaction of *P. brassicae* with different *Brassica* species and to identify the genes mediating clubroot resistance. Transcriptome analysis identified several genes involved in phytohormone and glucosinolate biosynthetic pathways, cell wall modification, and plant-pathogen interactions, as well as the genes pertaining to calcium signaling and receptor kinases, and those involved in pathogenesis and *Brassica-P. brassicae* interaction [10,11,12,15,17,18,24,25]. Proteomics studies reported several differentially abundant proteins (DAPs) involved in the regulation of phytohormones at early and late stages of infection by *P. brassicae* in *B. rapa* [14,21]. DAPs from the cytokinin (CK) signaling pathway were identified during the secondary stage of infection in *B. napus* and *B. rapa* [16,20], while Su et al. [26] detected proteins from the brassinosteroid (BR) signaling pathway. Beyond those, elements of MAPK signaling have been reported in *B. rapa* clubroot resistance through the gene *Rcr1*. Lastly, global proteome analysis by Adhikary et al. [19,27] reported proteins related to primary and secondary metabolism, calcium signaling, reactive oxygen species (ROS) scavenging, lignin biosynthesis, phytohormones, and dehydrins, indicating that mechanisms of resistance are likely multifaceted.

Transcriptome data provide a snapshot of the mRNA as well as non-coding RNAs in a cell at a given time point under specific environmental conditions, while proteome data provide information of the molecules resulting from the transcripts, offering information closer to the biological function of the genes. It is well known that not all mRNAs are necessarily transcribed into protein due to post-transcriptional modification, and not all proteins may be biologically active due to post-translational changes [28]. Thus, the expression of mRNA may not necessarily reflect the abundance of the biologically active proteins. Therefore, integration of transcriptome data with the proteome data could provide better insights into the molecular mechanisms involved in the control of the traits.

To date, most proteomics studies on clubroot resistance have been carried out using clubroot-resistant and susceptible cultivars or lines from segregating populations or doubled haploid progenies [16,20,21,22,27]. However, the use of diverse genetic material may identify not only the proteins exclusively involved in clubroot resistance but also the proteins affecting other traits. Here, we avoid these constraints through the use of clubroot-resistant and -susceptible near-isogenic lines (NILs). Using a comparative proteomics approach examining roots from clubroot-resistant (CR)- and clubroot-susceptible (CS)-NILs of *B. napus* in response to *P. brassicae* at three distinct stages of pathogenesis, we have identified a number of novel, putative targets that may contribute to clubroot resistance. Further, data integration with available transcriptome data [29] allows us to further resolve potential targets, while also generating a more wholistic, multi-omics understanding of the *B. napus-P. brassicae* pathosystem towards the development of enhanced clubroot resistance.

## 2. Results

### 2.1. P. brassicae Inoculation and Disease Symptom Assessment

**Figure 1:**
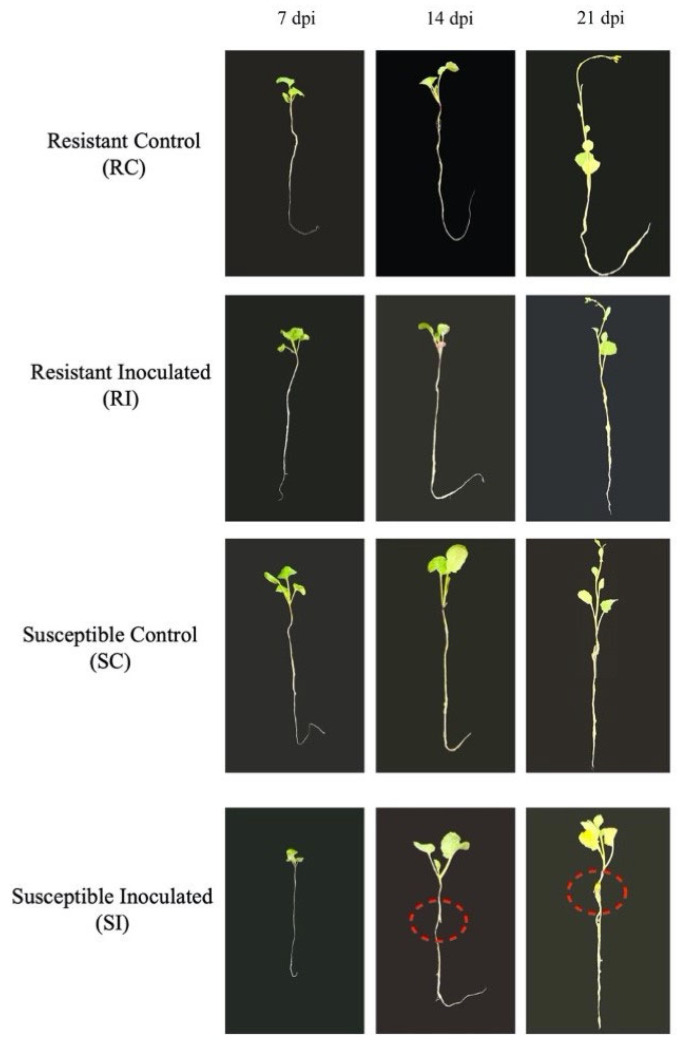
Root and shoot phenotypes of clubroot-resistant (CR) and -susceptible (CS) near-isogenic lines (NILs) of *Brassica napus* at 7, 14, and 21 days post-inoculation (dpi) with *Plasmodiophora brassicae* pathotype 3H and control (without inoculation). The dotted red circles show gall formation in the roots.

To assess clubroot disease onset and progression, we monitored gall formation in the roots of CR- and CS-NIL plants at 7, 14, and 21 dpi in both control and inoculated plants. Macroscopically, no visible symptoms were observed on the roots of both CR- and CS-NILs at 7 dpi; however, by 14 dpi, primary root had started to swell in inoculated CS-NILs, but no swelling symptoms were visible for the CR-NILs (Figure 1). At 21 dpi, the roots of the inoculated CS-NIL plants demonstrated clear root gall formation accompanied by distinct symptom manifestation (e.g., stunting, wilting, and yellowing) of aerial tissues in CS-NIL plants (Figure 1). Both the control and the inoculated CR-NILs did not show any sign of visible galls or any of the aforementioned symptoms (Figure 1).

### 2.2. Time-Course Proteomic Profiling of CR- and CS-NIL Roots Samples of B. napus

In this study, we performed quantitative proteome analysis of the *P. brassicae*-infected and uninfected roots at 7, 14, and 21 dpi. Before we proceeded with downstream analysis, we evaluated data quality both within biological replicates and between CR- and CS-NILs. Based on the Pearson correlation and principal component analysis (PCA), we observed that the biological replicates were consistent and tightly clustered within their respective groups with strong correlation (*r* > 0.95) (Figure S1A,B). Furthermore, following pathogen treatment, the inoculated CR (RI) and inoculated CS (SI) samples were clearly separated at the 95% confidence interval as the infection advanced to 14 and 21 dpi (Figure S1C). These results indicated that proteome data are reproducible and suitable for characterizing pathogenesis in the NILs. In the case of the transcriptome dataset, the principal component analysis also showed consistency among the biological replicates and good separation of the control and inoculated samples at both the time points; however, the separation was more pronounced at 14 dpi [29].

**Figure 2:**
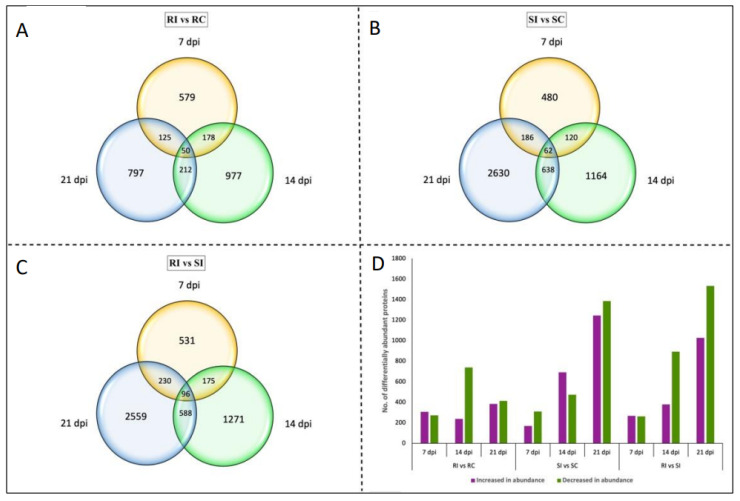
Differentially abundant proteins (DAPs) in roots of the clubroot-resistant (CR) and -susceptible (CS) *Brassica napus* near-isogenic lines (NILs) inoculated with *Plasmodiophora brassicae* pathotype 3H. The numbers indicate the number of DAPs significantly changing in abundance (*q*-value < 0.05) across three time points in a comparison of RI vs. RC (**A**), SI vs. SC (**B**), and RI vs. SI (**C**). (**D**) Variation in the number of proteins increasing or decreasing in abundance across the three comparisons at three time points. RI—inoculated resistant NILs; RC—uninoculated (control) resistant NILs; SI—inoculated susceptible NILs; SC—uninoculated (control) susceptible NILs; dpi—day post-inoculation.

Our proteome analysis identified a total of 6626 DAPs (*q*-value < 0.05), and this included 2353 and 4273 proteins in the CR- and CS-NILs, respectively (Figure 2). When compared with uninfected samples, 579, 977, and 797 DAPs were observed at 7, 14 and 21 dpi, respectively, in CR-NIL roots upon infection (Figure 2A). Among these, only 50 DAPs were common at all three time points (Table S1a), of which 12 showed an increased abundance and four showed a decreased abundance (Table S1a). In the case of the CS-NIL roots under infected and uninfected conditions, 480, 1164, and 2630 DAPs were observed at 7, 14, and 21 dpi, respectively (Figure 2B). Among these, 62 DAPs were detected across all three time points (Table S1b).

We next compared roots from the infected CR- and CS-NILs, where we detected 4361 DAPs (*q*-value < 0.05). Specifically, 531, 1271, and 2559 DAPs were observed at 7, 14, and 21 dpi, respectively (Figure 2C). Among these, 96 proteins were detected at all three time points, where 31 were observed to be increased and 13 decreased in abundances (Table S1c).

### 2.3. Temporal Changes in the CR- and CS-NIL Root Proteomes in Response to Pathogen Infection (RI vs. RC and SI vs. SC)

To best resolve how CR- and CS-NIL plants mount a response to pathogen challenge, we next examined our comparative quantitative proteomic CR- and CS-NILs data from a time point-specific perspective in order to best resolve time-dependent factors potentially contributing to resistance.

#### 2.3.1. 7 Days Post-Inoculation

**Figure 3:**
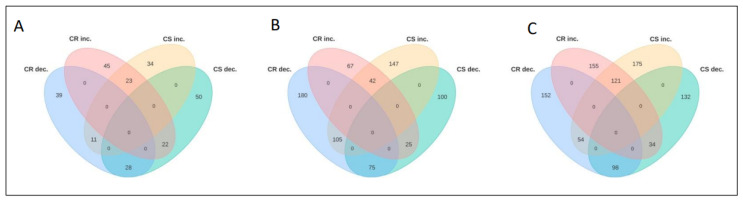
Temporal changes in the root proteome of the control and *Plasmodiophora brassicae* (pathotype 3H) inoculated clubroot-resistant (CR) and -susceptible (CS) *Brassica napus* near-isogenic lines (NILs). The numbers indicate the number of proteins that were significantly changing in abundance (*q*-value < 0.05): (**A**) 7 days post-inoculation (dpi); (**B**) 14 dpi; and (**C**) 21 dpi. CR—clubroot-resistant; CS—clubroot-susceptible; inc. and dec. represent increase and decrease, respectively.

At this stage, 45 and 34 DAPs were increased in abundance while 39 and 50 DAPs exhibited decreased abundance in the CR- and CS-NILs, respectively, in response to pathogen infection (Figure 3A). Among these, 22 DAPs were increased and 28 were decreased in abundance in both CR- and CS-NILs (Figure 3A), and this type of protein hereafter will be referred to as “shared proteins” (Table S2a). We also identified the proteins that exhibited an opposing pattern in the CR- and CS-NILs, i.e., either decreased in CR- but increased in CS-NILs, or vice-versa. These types of proteins will hereafter be referred to as “contrasting proteins”. A total of 33 DAPs were identified in the CR- and CS-NILs that belonged to this category (Table S2a).

Among the shared proteins, we found a number of interesting proteins, including a RECEPTOR-LIKE PROTEIN KINASE (BnaA01T0264300WE) and WRKY (BnaC07T0234800WE); both are potentially involved in the perception of pathogenesis-related signals that increased in abundance in both CR- and CS-NILs. On the other hand, proteins homologous to GEM-LIKE PROTEIN 5 (BnaA02T0047200WE), HSP70 PROTEIN 5 (BnaC08T0191000WE), and PEP CARBOXYLASE 3 (BnaC03T0502800WE) were decreased in abundance in both inoculated CR- and CS-NILs as compared to their controls. Among the contrasting proteins, the DAPs that increased in abundance in CR-NILs but decreased in the CS-NILs included stress-related proteins like DEHYDRIN ERD10 (BnaA07T0126200WE), PEROXYGENASE 3 (BnaA04T0204000WE), and RECEPTOR-LIKE KINASE (BnaC05T0048500WE), in addition to proteins involved in cell wall modification such as PECTIN ACETYLESTERASE 7 (PAE7, BnaA01T0022800WE). Other proteins such as PECTIN ESTERASE INHIBITOR 61 (PME61, BnaA02T0139800WE) and SENESCENCE-SPECIFIC CYSTEINE PROTEASE SAG12 (BnaA02T0292500WE) were decreased in CR-NILs but increased in the CS-NILs (Table S2a).

#### 2.3.2. 14 Days Post-Inoculation

When comparing the proteome profile at 14 dpi, we found 67 and 147 DAPs showing an increased abundance and 180 and 100 DAPs showing a decreased abundance in the CR- and CS-NILs, respectively (Figure 3B). Among these, 117 were shared proteins of which 42 showed an increase in abundance and 75 showed a decreased abundance in both CR- and CS-NILs (Table S2b). Among the 130 contrasting proteins, 25 showed increased abundance in the CR-NILs but decreased abundance in the CS-NILs, while 105 DAPs showed a decreased abundance in the CR-NILs but an increased abundance in the CS-NILs (Table S2b).

Among the shared proteins, BnaC06T0009500WE, a THIOREDOXIN H5 homolog, BnaC03T0109900WE (BASIC ENDOCHITINASE CHB4), and BnaA09T0013100WE (CHITINASE 10) all increased in abundance in both the CR- and CS-NILs. Among the contrasting proteins, the proteins homologous to ETHYLENE RESPONSIVE TRANSCRIPTION FACTOR 1A (ERF 1A, BnaA01T0006800WE), probable CALCIUM-BINDING PROTEIN CML49 (BnaA01T0296000WE), PEROXIDASE 44 (PER44, BnaA03T0495200WE), and PEROXIDASE 17 (PER17, BnaA04T0138000WE) showed an increased abundance in the CR-NILs but a decreased abundance in the CS-NILs. Contrary to this, the proteins involved in cell wall modifications such as PECTATE LYASE 5 (BnaA02T0167900WE) and XYLOGLUCAN-6-XYLOSYLTRANSFERASE 2 (XXT2, BnaA02T0256900WE), as well as protein homologous to RESPIRATORY BURST OXIDASE HOMOLOG D (RBOHD, BnaA02T0356200WE), showed decreased abundance in the CR-NILs, but an increased abundance in the CS-NILs.

#### 2.3.3. 21 Days Post-Inoculation

At 21 dpi, we identified 155 and 175 DAPs that were increased in abundance and 152 and 132 DAPs were decreased in abundance in the CR- and CS-NILs, respectively (Figure 3C). Among the shared proteins, 121 DAPs increased and 98 DAPs decreased in abundance in both CR- and CS-NILs (Table S2c). Among the 88 contrasting DAPs, 34 increased in abundance in CR-NILs but decreased in the CS-NILs, while 54 decreased in abundance in CR-NILs and increased in the CS-NILs (Table S2c).

Among the shared proteins, the stress-related proteins such as UNIVERSAL STRESS PROTEIN A-LIKE (BnaC06T0175200WE), THIOREDOXIN H5 (TRX5, BnaC06T0009500WE), and PEROXIDASE C2 (PRXC2, BnaA09T0252100WE) were increased in abundance in both CR- and CS-NILs. In the pool of contrasting proteins, the stress-related proteins homologous to PATHOGENESIS-RELATED MLP-LIKE PROTEIN 34 (BnaC06T0320700WE), CYSTEINE-RICH SECRETORY PROTEIN 9 (CRRSP9, BnaC04T0245900WE), CHITIN RECOGNITION PROTEIN ENDOCHITINASE CH25 (BnaC03T0326700WE), RECEPTOR-LIKE KINASE At1g51890 (BnaA06T0020600WE), the major intrinsic protein AQUAPORIN TIP1-1 (BnaA04T0224500WE) as well as the CALCIUM-BINDING PROTEIN CML49 (BnaA01T0296000WE) were increased in the CR-NILs but decreased in the CS-NILs.

### 2.4. Temporal Changes in the CR- and CS-NILs Root Proteomes upon Infection by P. brassicae (RI vs. SI)

We next compared inoculated CR- and CS-NIL roots, where we found a total of 96 DAPs of which 31 increased and 13 decreased in abundance in the CR-NILs at all three time points, 7, 14, and 21 dpi (Table S3). The proteins related to cell wall modifications such as XYLOGLUCAN ENDOTRANSGLUCOSYLASE/HYDROLASE PROTEIN 22 (BnaA02T0118100WE), EXPANSIN-A15 (BnaA02T0324300WE), putative TERPENOID SYNTHASE 7 (BnaA08T0101300WE), and DEFENSIN-LIKE PROTEIN 1 (BnaC02T0415700WE) were increased in abundance in the CR-NILs at all three time points (Table S3). Among the 13 proteins which showed a decreased abundance in the CR-NILs at these time points, proteins homologous to RETINOBLASTOMA-RELATED PROTEIN 1 (BnaA05T0375100WE) and TRICYCLENE SYNTHASE (TPS10, BnaC03T0219500WE) were observed.

Alternatively, a total of 52 proteins showed variable abundance changes over the time-course (Table S3). For example, some exhibited a decrease in abundance at 7 dpi but increased abundance at 14 and 21 dpi. These included probable 2-OXOGLUTARATE-DEPENDENT DIOXYGENASE AOP1 (BnaA01T0266700WE) potentially involved in glucosinolate biosynthesis, RALF-LIKE 22 (BnaA01T0304800WE) potentially involved in calcium mediated signaling, RPM1-INTERACTING PROTEIN 4 (BnaA03T0318600WE), LRR-RLK (BnaA05T0167600WE), and HYPERSENSITIVE-INDUCED RESPONSE PROTEIN 3 (BnaC05T0548200WE) involved in conferring resistance to infection by a pathogen (Table S3).

### 2.5. GO Enrichment of Differentially Abundant Proteins (DAPs)

**Figure 4:**
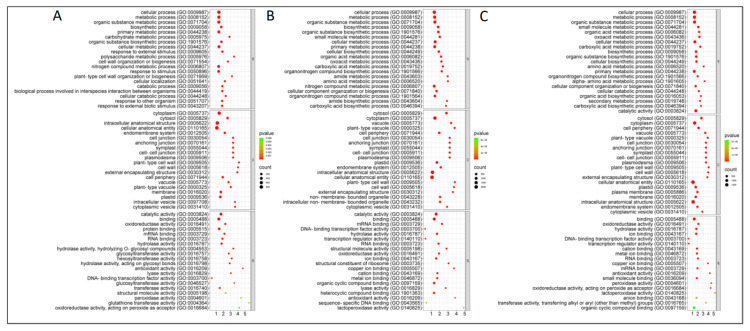
The top 20 significantly enriched gene ontology (GO) terms of each of the biological process (BP), molecular function (MF), and cellular components (CC) categories for the differentially abundant proteins (DAPs) identified from RI vs. RC (**A**), SI vs. SC (FDR < 0.05) (**B**), and RI vs. SI (**C**) comparisons in roots of *Brassica napus* near-isogenic lines (NILs) at 7, 14, and 21 days post-inoculation (dpi) with *Plasmodiophora brassicae* pathotype 3H. RI—inoculated-resistant NILs; RC—uninoculated (control)-resistant NILs; SI—inoculated-susceptible NILs; SC—uninoculated (control)-susceptible NILs.

To identify the broader biological processes of the quantified DAPs, we performed the GO enrichment analysis (Figure 4). In the CR-NILs (inoculated vs. control), 404 GO terms were significantly enriched (FDR < 0.05) by 21 dpi; among these, 257 terms belonged to the biological process (BP), 43 belonged to the molecular function (MF), and 104 belonged to the cellular components (CC) category (Table S4a). The top 20 significant GO terms in each of the BP and MF categories included response to external stimulus (GO:0009605), biological processes involved in interspecies interaction between organisms (GO:0044419), response to other organism (GO:0051707), response to external biotic stimulus (GO:0043207), response to biotic stimulus (GO:0009607), oxidoreductase activity (GO:0016491), antioxidant activity (GO:0016209), peroxidase activity (GO:0004601), amongst others.

In the CS-NILs (SI vs. SC comparison), 363, 107, and 94 GO terms belonging to the BP, MF, and CC categories, respectively, were significantly (FDR < 0.05) enriched in the CS-NILs by 21 dpi (Table S4b). The top GO terms in the BP category included the organic substance metabolic process (GO:0071704), primary metabolic process (GO:0044238), amino acid metabolic process (GO:0006520), and others. The top significant GO terms of the MF category included oxidoreductase activity (GO:0016491), ion binding (GO:0043167), cation binding (GO:0043169), and lyase activity (GO:0016829).

While analyzing the DAPs from the RI vs. SI comparison, a total of 685 GO terms (419, 134, and 132 related to BP, MF, and CC category, respectively) were significantly enriched (FDR < 0.05) as the infection progressed from 7 to 21 dpi (Table S4c). The top significant (FDR < 0.05) terms of the BP category included the organic substance metabolic process (GO:0071704), primary metabolic process (GO:0044238), and amino acid metabolic process (GO:0006520), and those of the MF category included oxidoreductase activity (GO:0016491), ion binding (GO:0043167), cation binding (GO:0043169), and lyase activity (GO:0016829); these GO terms were also enriched in the SI vs. SC comparison. The top 20 significant GO terms from each of the category are represented in Figure 4.

### 2.6. Identification of Putative DAPs Involved in Clubroot Resistance

#### 2.6.1. Phytohormone-Mediated Signaling

When uninoculated and inoculated CR- and CS-NILs were compared to their respective controls (RI vs. RC and SI vs. SC), we identified 14 DAPs related to auxin and five DAPs associated with salicylic acid (SA) regulation and three of them (probable PEROXYGENASE 3, BnaA04T0204000WE; TRANSCRIPTION FACTOR TGA6, BnaA03T0338500WE; UDP-GLYCOSYLTRANSFERASE 74C1, BnaA05T0120800WE) showed increased abundance in the CR-NILs and decreased in abundance in the CS-NILs. Two DAPs (protein DR1 HOMOLOG, BnaA03T0047100WE, UDP-GLYCOSYLTRANSFERASE 74C1, and BnaA05T0120800WE) showed decreased abundance in both CR- and CS-NILs (Table S5a). In the case of jasmonic acid (JA), we identified 12 DAPs, and among them, three showed an increased abundance, six showed decreased abundance in both the CR- and CS-NILs, and the remaining three showed a contrasting abundance pattern in the CR- and CS-NILs. Two of the three contrasting DAPs for JA were homologous to 3-KETOACYL-COA THIOLASE 2, and PEROXISOMAL (PED1, BnaA05T0111200WE, and BnaA04T0202400WE). In addition to the above, we also detected four DAPs associated with ethylene (ET) regulation. Three of these showed increased accumulation upon inoculation in both CR- and CS-NILs with a higher level of abundance in the CS-NILs (Table S5a).

When the RI and SI root proteomes were compared, we detected two DAPs associated with SA and JA regulation. The DAP related to SA regulation (BnaA03T0338500WE) increased in abundance in the CR-NILs as the infection progressed from 7 to 21 dpi. In contrast, the JA-related DAP homologous to TRICYCLENE SYNTHASE (TPS10, BnaC03T0219500WE) decreased in abundance in the CR-NILs at all three time points following infection (Table S5b).

#### 2.6.2. Calcium-Mediated Signaling

Based on comparisons between uninoculated and inoculated CR- and CS-NILs as compared to their respective controls (RI vs. RC and SI vs. SC), we identified seven DAPs associated with calcium-mediated signaling. Three of these DAPs were homologous to calcium-binding proteins CML49 (BnaA01T0296000WE), MLO-LIKE PROTEIN 6 (BnaA09T0149900WE), and GLUTAMATE DECARBOXYLASE 2 (BnaC06T0348200WE), and they increased in abundance in the CR-NILs but decreased in abundance in the CS-NILs (Table S5a). Similarly, when comparisons were made between RI vs. SI, three DAPs related to VACUOLAR-SORTING RECEPTOR 6 (BnaA09T0374500WE), RALF-LIKE 22 (BnaA01T0304800WE), and RALF-LIKE 1 (BnaA08T0299900WE) were identified. Two of these (VACUOLAR-SORTING RECEPTOR 6 and RALF-LIKE 22) showed an increase in abundance in the CR-NILs as the infection progressed from 7 to 21 dpi (Table S5b).

#### 2.6.3. Reactive Oxygen Species-Mediated Signaling

Upon inoculation of the CR- and CS-NILs and comparison with their respective controls (RI vs. RC and SI vs. SC), 24 DAPs were detected (*q*-value < 0.05) that were related to ROS. Thirteen of these proteins demonstrated a similar increasing trend in abundance in both CR- and CS-NILs while eight showed a contrasting trend in their abundance in the CR- and CS-NILs. For instance, proteins homologous to PEROXYGENASE 3 (PXG3, BnaA04T0204000WE), CATALASE 2 (CAT2, Bnascaffold286T0031400WE), GLUTATHIONE-S-TRANSFERASE U12 (GSTU12, BnaA02T0183300WE), PEROXIDASE 2 (PER2, BnaA09T0647200WE), and L-GULONOLACTONE OXIDASE 2 (GULLO2, BnaC04T0010900WE) were increased in CR-NILs and were observed to be decreased in abundance in the CS-NILs (Table S5a). Notably, one of the proteins homologous to the ATP-dependent Clp protease proteolytic subunit (BnaA01T0100800WE) was consistently increased in abundance at all three time points in the inoculated CR-NILs in contrast to the inoculated CS-NILs (Table S5b).

#### 2.6.4. Glucosinolates

When compared between uninoculated and inoculated CR- and CS-NILs as compared to their respective controls (RI vs. RC and SI vs. SC), five DAPs involved in glucosinolate biosynthesis were identified. Two of the proteins, related to BETA-GLUCOSIDASE 19 (BGLU19, BnaC05T0118300WE) and NITRILE SPECIFIER PROTEIN 2 (NSP2, BnaC05T0414100WE), were increased in CR-NILs but decreased in CS-NILs (Table S5a). Similarly, when comparisons were made between RI and SI, we identified three DAPs (*q*-value < 0.05) that were homologous to NITRILE SPECIFIER PROTEIN 5 (NSP5, BnaC02T0465400WE), 2-OXOGLUTARATE-DEPENDENT DIOXYGENASE AOP1 (BnaA01T0266700WE), and NSP2 (BnaC05T0414100WE), and their abundance was increased in the inoculated CR-NILs at two or more points (Table S5b).

#### 2.6.5. Resistance Proteins

When comparisons were made between uninoculated and inoculated CR- and CS-NILs as compared to their respective controls (RI vs. RC and SI vs. SC), we found six DAPs related to disease resistance. Among these, four proteins homologous to (+)- NEOMENTHOL DEHYDROGENASE SDR1 (BnaA10T0002900WE), ENHANCED DISEASE RESISTANCE 2-LIKE (EDR2L, BnaA03T0055600WE), and PROTEIN YLS9 (BnaA08T0237900WE, BnaC05T0143800WE) were increased in abundance in the CR-NILs but decreased in the CS-NILs (Table S5a). We also found two DAPs homologous to RPM1-INTERACTING PROTEIN 4 (RIN4, BnaA03T0318600WE) and SDR1 (BnaA10T0002900WE) showing an increased abundance in the inoculated CR-NILs at one or more time points (Table S5b).

#### 2.6.6. General Stress-Related Proteins

While comparing the root proteomes of the inoculated CR-NILs and inoculated CS-NILs with their respective controls (RI vs. RC and SI vs. SC), we found 23 significant DAPs associated with general stress responses (Table S5a), nine of which increased in abundance in both CR- and CS-NILs. On the other hand, five DAPs increased in abundance in the CR-NILs but decreased in abundance in the CS-NILs, and this included the proteins related to PEROXYGENASE 3 (PXG3, BnaA04T0204000WE), DEHYDRIN ERD10 (BnaA07T0126200WE), SERINE/ARGININE-RICH SPLICING FACTOR SR45A (BnaA08T0160100WE), TRANSCRIPTION FACTOR RF2B (BnaC03T0580600WE), and ENDOCHITINASE (CH25, BnaC03T0326700WE).

In the RI vs. SI comparison, we found three DAPs, viz. PATELLIN-1 (BnaC02T0252500WE), DEFENSIN-LIKE PROTEIN 1 (BnaC02T0415700WE), and putative VACUOLAR PROTEIN SORTING-ASSOCIATED PROTEIN 13A (BnaC08T0102600WE), to be associated with general stress responses. All these DAPs showed a consistently increased abundance at all three time points in the inoculated CR-NILs (Table S5b).

#### 2.6.7. Lipid Metabolism

In response to the pathogen infection, 17 proteins related to lipid biosynthesis or degradation were differentially accumulated (*q*-value < 0.05) in the CR- and CS-NILs as compared to their respective controls (RI vs. RC and SI vs. SC). Among these, three proteins homologous to LIPID DEGRADATION GDSL ESTERASE/LIPASE (BnaA06T0007800WE, BnaA08T0187900WE, and BnaC06T0071100WE) were decreased in abundance in both CR- and CS-NILs (Table S5a). On the other hand, three DAPs homologous to LIPID PHOSPHATE PHOSPHATASE GAMMA (BnaA02T0009800WE), ACYL-ACTIVATING ENZYME 4 (AEE4, BnaA07T0363700WE), and FATTY ACID AMIDE HYDROLASE (FAAH, BnaC03T0458600WE) showed an increased abundance in the inoculated CR-NILs but a decreased in abundance in the inoculated CS-NILs.

While comparing the inoculated CR-NILs and CS-NILs (RI vs. SI), we identified two DAPs (*q*-value < 0.05) that were homologous to fatty acid biosynthesis [12-OXOPHYTODIENOATE REDUCTASE 1 (OPR1, BnaC02T0289200WE)] and fatty acid degradation [ENOYL-COA DELTA ISOMERASE 3 (ECI3, BnaC08T0081300WE)]. Notably, the OPR1 increased in abundance in the CR-NILs at all three time points, while ECI3 increased in abundance in the CR-NILs at 7 dpi but decreased at 14 and 21 dpi (Table S5b).

#### 2.6.8. Cell Wall Modifications

In response to the pathogen infection, we identified 34 DAPs (*q*-value< 0.05) to be associated with cell wall modifications, including those related to the biosynthesis of pectin, lignin and cellulose. Seven of these proteins, related to XYLOGLUCAN ENDOTRANSGLUCOSYLASE/HYDROLASE PROTEIN 22 (XTH22, BnaA02T0118100WE), CAFFEOYLSHIKIMATE ESTERASE (CSE, BnaA03T0206500WE), ALPHA-GLUCOSIDASE 2 (GCS2, BnaA07T0098300WE), PEROXIDASE C2 (PRXC2, BnaA09T0252100WE), probable GALACTURONOSYLTRANSFERASE 4 (GAUT4, BnaA02T0309700WE), GLYCOSYLTRANSFERASE LIKE FAMILY 2 (BnaA03T0110700WE), and CAFFEIC ACID 3-O-METHYLTRANSFERASE (COMT1, BnaC07T0166400WE) exhibited increased abundance in both inoculated CR- and CS-NILs (Table S5a). Six proteins, related to CSE (BnaA04T0244300WE), PEROXIDASE 44 (PER44, BnaA03T0495200WE), PER17 (BnaA04T0138000WE), SHIKIMATE-O-HYDROXYCINNAMOYLTRANSFERASE (HST, BnaC06T0472200WE), PER2 (BnaA09T0647200WE), and ENDOGLUCANASE 7 (KOR2, BnaA02T0157700WE) were increased in abundance in the inoculated CR-NILs but decreased in abundance in the CS-NILs (Table S5a).

When comparing inoculated CR-NILs with the inoculated CS-NILs, we detected eight DAPs (*q*-value < 0.05) related to cell wall modifications. Five of them including XTH22 (BnaA02T0118100WE), EXPANSIN-A15 (EXPA15, BnaA02T0324300WE), PUTATIVE CAFFEOYL-COA O-METHYLTRANSFERASE (At1g67980, BnaC08T0082100WE), probable POLYGALACTURONASE (At1g80170, BnaC06T0268800WE), and 21kDa protein (Bnascaffold1556T0003100WE) showed increased abundance in the inoculated CR-NILs as the infection progressed from 7 to 21 dpi (Table S5b).

### 2.7. Multi-Omics Integration: Transcriptomics and Proteomics

To further contextualize our proteomic findings, we compared them with our recently published transcriptome datasets [29]. For this, we assessed the correlation between transcript regulation and protein abundance at 7 and 14 dpi in both CR- and CS-NILs. In the CR-NILs, no significant correlation was observed at 7 dpi; however, a positive correlation was observed at 14 dpi (Pearson *r* = 0.910; *p* < 0.0000; Spearman *r* = 0.649; *p* < 0.000; slope = 0.923; R^2^ = 0.828) (Figure S2). In the CS-NILs, a moderate but statistically significant positive correlation was observed at 7 dpi (Pearson *r* = 0.494; *p*-value = 0.0041; Spearman *r* = 0.543, *p*-value = 0.0016), and the correlation became stronger at 14 dpi (Pearson *r* = 0.832; *p* < 0.000; Spearman *r* = 0.676, *p* < 0.000; slope = 0.702, R^2^ = 0.692). While comparing the inoculated CR- and CS-NILs (RI vs. SI), no significant correlation was observed at 7 dpi; however, a strong and positive correlation was observed at 14 dpi (Pearson *r* = 0.626, *p* = 0.0000); Spearman *r* = 0.749, *p* < 0.0000; slope = 0.424; R^2^ = 0.392).

**Figure 5:**
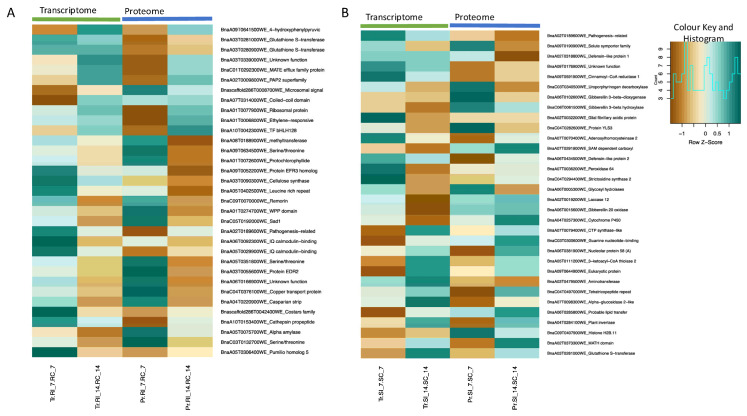
Differentially abundant transcripts (genes) and proteins in roots of the clubroot-resistant (CR) and -susceptible (CS) *Brassica napus* near isogenic lines (NILs) in response to infection by *Plasmodiophora brassicae* pathotype 3H. (**A**) Log_2_ fold changes of the transcripts and proteins at 7 and 14 dpi when comparing the inoculated versus uninoculated samples of the resistant NILs (RI vs. RC). (**B**) Log_2_ fold changes of transcripts and proteins at 7 and 14 dpi when comparing inoculated vs. uninoculated samples of the susceptible NILs (SI vs. SC). Transcripts that are differentially abundant at one or more time points (*q*-value < 0.05) are presented in the heatmaps. Gene expression and protein abundance values are displayed as row z-scores for visualization. A z-score of 0 indicates average expression, positive values represent relative upregulation (dark green, higher abundance on the heatmap), and negative values represent relative downregulation (dark brown, lower abundance on the heatmap). The data below the light green and blue bar indicate transcriptome and proteome data, respectively. RI—resistant inoculated; RC—resistant uninoculated (control); SI—susceptible inoculated; SC—susceptible uninoculated (control); 7 and 14 indicates 7 and 14 days post-inoculation; Tr—transcriptome; Pr—proteome.

From integrated analysis of the transcriptome and proteome datasets of the inoculated CR-NILs (RI vs. RC), we found 33 transcripts that share a common pattern of differential expression at one or more time points. Of these, 11 were significantly increased and 22 were significantly decreased (*q*-value < 0.05) in abundance at one or more time points (Figure 5A; Table S6a). Gene transcripts related to *4-HYDROXYPHENYLPYRUVATE DIOXYGENASE* (BnaA09T0641500WE) and *GLUTATHIONE S-TRANSFERASE* (BnaA03T0281000WE, BnaA03T0280900WE) were consistently increased in abundance (>1.8 log_2_ folds; *q*-value < 0.05) at one or more time points in both studies (Table S6a). Similarly, other transcripts encoding *COILED-COIL DOMAIN* (BnaA07T0314000WE), *ETHYLENE-RESPONSIVE TRANSCRIPTION FACTOR 1A* (BnaA01T0006800WE), and *LIPID PHOSPHATE PHOSPHATASE GAMMA* (BnaA02T0009800WE) were also significantly increased (*q*-value < 0.05) in abundance at one or more time points in both studies.

In the CS-NILs (SI vs. SC), 32 transcripts shared a common pattern in both the transcriptome and proteome studies at one or more time points. Of these, 13 transcripts were significantly increased (*q*-value < 0.05) and 19 transcripts were significantly decreased (*q*-value < 0.05) in abundance at one or more time points (Figure 5B; Table S6b). Transcripts related to *GLUTATHIONE S-TRANSFERASE F2* (BnaA03T0281000WE), *MATH DOMAIN* (BnaA02T0373300WE), and *PLANT INVERTASE/PECTIN METHYLESTERASE INHIBITOR* (BnaA04T0284100WE) were significantly increased in abundance at one or more time points (>1.5 log_2_ folds; *q*-value < 0.05) in both studies (Table S6b). In contrast, transcripts related to *PATHOGENESIS-RELATED PROTEIN* (BnaA02T0189600WE), *SODIUM SOLUTE SYMPORTER FAMILY* (BnaA09T0190900WE), and *DEFENSIN-LIKE PROTEIN* (BnaA02T0318800WE, BnaA06T0434500WE) were significantly decreased in both transcriptome and proteome studies at 14 dpi.

**Figure 6:**
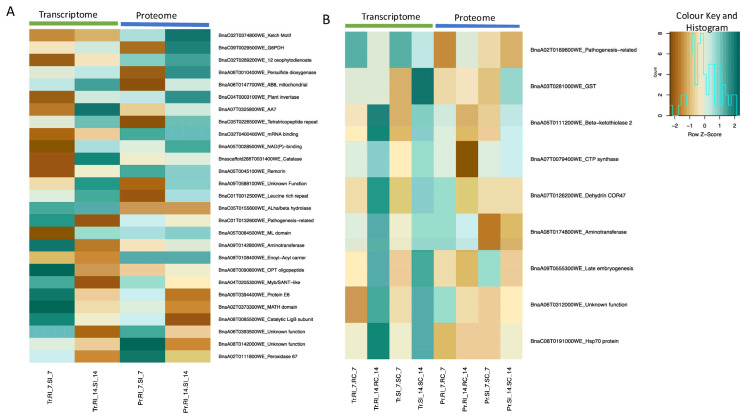
Differentially abundant transcripts (genes) and proteins in roots of the clubroot-resistant (CR) and -susceptible (CS) *Brassica napus* near-isogenic lines (NILs) in response to infection by *Plasmodiophora brassicae* pathotype 3H. (**A**) Log_2_ fold changes of the transcripts and proteins at 7 and 14 dpi when comparing the inoculated resistant NILs versus inoculated susceptible NILs (RI vs. SI). (**B**) Transcripts and proteins across all time points for a comprehensive comparison. The heatmap shows log_2_ fold changes of common transcripts and proteins at 7 and 14 dpi when comparing inoculated vs. uninoculated samples across both resistant and susceptible NILs. Transcripts that are differentially abundant at one or more time points (*q*-value < 0.05) are presented in the heatmaps. Gene expression and protein abundance values are displayed as row z-scores for visualization. A z-score of 0 indicates average expression, positive values represent relative upregulation (dark green, higher abundance on the heatmap), and negative values represent relative downregulation (dark brown, lower abundance on the heatmap). The data below the light green and blue bars indicate transcriptome and proteome data, respectively. RI—resistant inoculated; RC—resistant uninoculated (control); SI—susceptible inoculated; SC—susceptible uninoculated (control); 7 and 14 indicates 7 and 14 days post-inoculation; Tr—transcriptome; Pr—proteome.

When transcriptome and proteome data were integrated and compared between the inoculated CR- and CS-NILs (RI vs. SI), 48 transcripts and proteins were observed to be differentially abundant at one or more time points (Table S6c), of which 27 transcripts showed a consistent pattern of differential abundance (*q*-value < 0.05) at one or more time points (Figure 6A). Among these, 14 transcripts exhibited increased abundance and 13 showed a decrease in abundance at one or more time points in the CR-NILs (Figure 6A; Table S6c). Some of the key transcripts sharing a common pattern included *REMORIN* (BnaA05T0045100WE), *CATALASE-RELATED IMMUNE RESPONSIVE* (Bnascaffold286T0031400WE), *KECH MOTIF* (BnaC02T0374800WE), *12-OXOPHYTODIENOATE REDUCTASE 1* (BnaC02T0289200WE), *PLANT INVERTASE/PECTIN METHYLESTERASE INHIBITOR* (BnaC04T0003100WE), and *GLUCOSE-6-PHOSPHATE DEHYDROGENASE* (BnaC09T0029500WE), and these transcripts showed an increased abundance (*q*-value < 0.05) at 14 dpi (Table S6c).

Similarly, when comparing pathogen-inoculated and uninoculated NILs at both time points (7 and 14 dpi) in both genotypes, nine transcripts were significantly differentially abundant (*q*-value < 0.05) at one or more time points in both transcriptome and proteome levels (Figure 6B). Several transcripts, including *GLUTATHIONE S-TRANSFERASE F2* (BnaA03T0281000WE) and PATHOGENESIS-RELATED PROTEIN (BnaA02T0189600WE), were differentially abundant (Table S6d). Notably, the gene *GLUTATHIONE S-TRANSFERASE F2* (BnaA03T0281000WE) was significantly upregulated at 7 and 14 dpi in the CR-NILs in both transcriptome and proteome studies. In the CS-NILs, the gene was also significantly upregulated at both 7 and 14 dpi at the proteome level; however, at the transcriptome level, expression of this gene was significantly increased only at 14 dpi (Table S6d).

Based on gene ontology (GO) enrichment analysis, within the molecular function, categories involving oxidoreductase activity, metal ion binding, and cinnamoyl-CoA reductase activity were significantly enriched (adjusted *p*-value < 0.05) (Table S7A). Within the biological process, categories involving response to nucleus organization, negative regulation of circadian rhythm, and nitrile biosynthetic process were significantly enriched (adjusted *p*-value < 0.05). (Table S7B). In terms of the KEGG pathway, the category involving metabolic pathways is significantly enriched (adjusted *p*-value < 0.05) (Table S7C).

## 3. Discussion

Advances in high-throughput molecular omics technologies have facilitated a holistic investigation of the complex interplay between *B. napus* and *P. brassicae* through genomics, transcriptomics, proteomics, and metabolomics [19,27,30,31,32]. Proteomics alone has also been used to elucidate the molecular basis of clubroot resistance and susceptibility in *B. napus* [16,19,20,22]. However, few studies have been carried out using NILs to characterize the *P. brassicae* pathogenesis in *B. napus* at the proteome level. In this study, we carried out a comparative proteomics study using clubroot-resistant and -susceptible *B. napus* NILs and integrated this information with the data obtained from a transcriptomics study using the same plant materials. Our results were based on four independent biological replicates per treatment where each biological replicate included 21 pooled root samples. This approach has both strengths and limitations. On the one hand, pooling reduces the loss of low expressed genes [33], enhances the representativeness of a broader population, reduces the biological variation [34], potentially provides a robust estimate of the average resistance response and facilitates the identification of major expression shifts linked to host resistance or susceptibility [19]. On the contrary, this approach may introduce the risk of unequal contribution of RNA from individual pooled samples, especially if RNA yield differs across the pooled samples. Furthermore, it may also mask molecular variability among the individual plants, particularly if some of the individuals show a stronger or weaker resistance and which may average out the molecular signals. In this study, our objective was not to capture the plant-to-plant variations within the CR- or CS-NILs, it was rather to characterize the resistance pattern at a population level. In addition, variation in resistance between the individual plants of the CR- and CR-NILs was minimum. However, to further minimize the bias during sample collection, we carefully used the roots of comparable size at similar developmental stages; thus, the experimental materials can be considered suitable for transcriptome and proteome analysis.

From this study, we identified several proteins that were differentially abundant in response to pathogen infection and identified the proteins uniquely abundant in the CR-NILs. While most observed proteins and metabolic pathways are like the previous reports in *B. napus*, *B. oleracea* and *B. rapa* [19,22,23], our current study using NILs has provided additional evidence for the involvement of specific metabolic pathways and proteins in the resistance mechanism.

### 3.1. Perception of Infection Signals

Plants rely on a two-pronged immune system, PTI and ETI, to defend against pathogen infection. In phase 1, they employ PRRs to detect pathogen-associated molecular patterns (PAMPs) and initiate PAMP-triggered immunity (PTI). In the first phase, the plant perceives the pathogen attack and responds through PTI and employs a broad spectrum of transmembrane signaling proteins, which are essential for the recognition of the pathogen and activation of innate host-immune responses [35,36]. When the pathogen overcomes the first level of defense and delivers effector molecules inside the host cells, in phase II, the host plant recognizes them and activates the effector-triggered (ETI) immune system. By examining a combination of pathogen-inoculated and uninoculated CR- and CS-NIL roots, we identified a number of putative signaling proteins potentially important to clubroot resistance, including an increased abundance of several receptor kinases or PRR proteins homologous to LRR receptor-like serine/threonine-protein kinase in the CR-NILs in response to pathogen attack. These receptor proteins contain an extracellular domain to identify the pathogen signals and activate PTI [37]. Calcium ions (Ca^2+^) play a vital role in PTI as well as in ETI [38,39]. For Ca-mediated signaling, we found an increased abundance of CML49 (BnaA01T0296000WE), RALFL22 (BnaA01T0304800WE), and RALF1 (BnaA08T0299900WE) at two or more time points in the CR-NILs. RALF1 and RALF22 interact with receptor kinase FERONIA (FER) localized in the plasma membrane [40,41]. Another protein IQD1 (BnaA01T0298100WE), a CALMODULIN BINDING (CAB) nuclear protein, was highly increased (*q*-value < 0.05) in the CR-NILs and decreased in the CS-NILs. IQD1 positively regulates glucosinolate accumulation, in response to biotic stresses [42]. This observation of Ca^2+^ signaling proteins aligns perfectly with earlier studies in *B. napus,* where proteins related to CML and CAB showed differential abundance in the early and late stages of pathogenesis [19,27]. We also observed an increase in abundance of three ubiquitin-dependent proteins, 26S PROTEASOME NON-ATPASE REGULATORY SUBUNIT 2 HOMOLOG B (RPN1B, BnaA01T0006100WE), E3 UBIQUITIN protein ligase (RGLG2, BnaA07T0290200WE), and UBIQUITIN CARBOXYL-TERMINAL HYDROLASE 12 (UBP12, BnaC02T0487200WE) in the CR-NILs. Earlier studies with *Arabidopsis* demonstrated that ubiquitination of pattern recognition receptor FLS2 leads to the activation of signaling pathways [43,44,45], and the regulatory role of ubiquitin-dependent proteins in defense response against *P. brassicae* in *B. rapa* has also been reported by Song et al. [23]. This suggests that the ubiquitin-mediated signaling pathways may play a potential role in the *P. brassicae*-*B. napus* pathosystem.

### 3.2. Phytohormone-Mediated Signaling Implications

Auxin response factors (ARFs) bind to the auxin-responsive elements (AuxRe) and positively regulate the auxin-mediated transcriptional responses [46]. ARFs target the genes regulating the homeostasis of auxins. ARF7 is reported to regulate the auxin-dependent differential growth in the hypocotyls of *Arabidopsis* [47]. We found a decreased abundance of ARF7 (BnaA02T0087300WE) in the CS-NILs, which might be associated with the reduction in lateral root formation favoring the gall formation (Table S5a). Similar results have been reported in *A. thaliana* in response to *P. brassicae* infection [48]. We also observed an increased abundance of a protein orthologous to *A. thaliana* ILL2 (BnaA10T0122400WE) in the CR-NILs (Table S5a). The ILL2 (IAA-amino acid hydrolase ILR1-like 2) encodes IAA-amino acid hydrolase possessing a broad range of substrate specificity [49] and functions in the inactivation or inhibition of the phytohormone auxin. This suggests that BnaA10T0122400WE could play a role in auxin inhibition in the CR-NILs. It is also known that the direction and distribution of auxin in the roots are regulated by *AUXIN EFFLUX CARRIER COMPONENT* (*PIN*) genes [50]. Robin et al. [51] found an increased transcription of BrPIN1 in dividing cells of *B. rapa* roots during cortical infection by *P. brassicae* and gall formation. In this study, we also observed an increase in abundance of the protein orthologous to AtPIN1 (BnaA07T0334800WE) in the CS-NILs but decreased in abundance in the CR-NILs (Table S5a). Based on this, it is likely that this protein may play a crucial role in gall formation in the susceptible *B. napus* plants.

We observed a contrasting pattern of accumulation for TGA6-related protein BnaA03T0338500WE in the CR- and CS-NILs (Table S5a). The TGA transcription factor family regulates the transcription of SA-responsive genes through interactions with *NON-EXPRESSOR OF PATHOGENESIS RELATED 1* (*NPR1*) gene [52]. Moreover, it has been reported that TGA6 is required for the induction of the *PATHOGENESIS RELATED 1* (*PR1*) gene in the presence of SA and that it also plays a role in the positive regulation of the systemic acquired resistance [53]. Additional investigation into the precise role(s) of the TGA6-related protein (BnaA03T0338500WE) in mediating clubroot resistance is warranted.

In the case of JA, *TRICYCLENE SYNTHASE 10* (*TPS10*) gene is reported to be involved in the jasmonate defense signaling pathway [54]. A higher accumulation of JA in susceptible *B. napus* plants at 14 to 28 dpi has been previously reported [53,55]. In this study, we observed a decreased abundance of the protein related to TPS10 (BnaC03T0219500WE) in the CR-NILs at all three time points (Table S5b). *ETHYLENE RESPONSIVE FACTORS* (*ERFs*) can be either activated or repressed in the host plant in response to pathogen infection [56]. We found an increased abundance of ERF1A (BnaA01T0006800WE) in the CR-NILs but a decreased abundance in the CS-NILs upon infection when compared to their respective uninoculated controls (Table S5a). Our results suggest a potential role for this gene in mediating clubroot resistance.

### 3.3. Resistance Proteins (R-Proteins)

The enhanced disease resistance 2 (EDR2) protein limits the initiation of cell death during plant−pathogen interactions [57]. The HAIRPIN-INDUCED FAMILY protein (YLS9) has been reported to play a role in defense mechanism against *Sclerotinia sclerotiorum* in *B. napus* [58]. The gene *(+)- NEOMENTHOL DEHYDROGENASE* (*SDR1*) plays a role in basal resistance against pathogens [59,60]. In this study, we identified four putative proteins related to EDR2-like (BnaA03T0055600WE), YLS9 (BnaA08T0237900WE, BnaC05T0143800WE), and SDR1 (BnaA10T0002900WE), which exhibited an increase in abundance in the CR-NILs but a decrease in abundance in the CS-NILs (Table S5a), implying that they might be involved in *P. brassicae−B. napus* interaction. We also identified the protein orthologous to RIN4, which showed an increased abundance in the inoculated CR-NILs when compared with the inoculated CS-NILs as the infection progressed from 7 to 21 dpi (Table S5b). The RIN4 (*RPM1-INTERACTING PROTEIN 4*) gene is known to target various bacterial effectors either directly or indirectly, as well as to activate R-proteins [61,62]. We have also previously reported the detection of 13 lncRNAs regulating *RIN4* [63]. Taken together with our current observations, it may be inferred that *RIN4* might be involved in modulating the innate immunity in *B. napus* against *P. brassicae*.

### 3.4. Glucosinolate Metabolism

The hydrolysis products of glucosinolates are known to play various roles in plant defense responses against bacterial and fungal pathogens [64]. The NITRILE-SPECIFIER PROTEIN (NSP) having 30–45% sequence homology with the EPITHIOSPECIFIER proteins (ESPs) activates the myrosinase-catalyzed degradation of glucosinolates to nitriles [65]. In this study, we identified the proteins orthologous to NSP2 (BnaC05T0414100WE) (Table S5a) and NSP5 (BnaC02T0465400WE) (Table S5b), which exhibited increased abundance in the inoculated CR-NILs. We also observed an increase in abundance of another protein orthologous to 2-OXOGLUTARATE-DEPENDENT DIOXYGENASE AOP1 (BnaA01T0266700WE) in the CR-NILs as the infection progressed from 7 to 21 dpi (Table S5b). It is reported in the literature that AOP1 is involved in the glucosinolate biosynthetic pathway [66], suggesting a role for glucosinolates in clubroot resistance.

### 3.5. Multi-Omics Analysis Reveals ROS Signaling and Cell Wall Reinforcement as a Likely Strategy for P. brassicae Defense

During the interaction between *B. napus* and *P. brassicae*, cell wall reinforcement by lignin accumulation is a key strategy underlying host resistance [67]. Earlier omics studies identified several genes related to *PLANT INVERTASE/PECTIN METHYLESTERASE INHIBITOR*, lignin biosynthesis, remorins, and peroxidases [19,27] that are involved in maintaining cell wall integrity and modulating plant growth and defense mechanisms. In this study, we found several genes related to *PECTIN METHYLESTERASE INHIBITOR*, *KELCH MOTIF*, and *LACCASE*, and the differential abundance of these genes was increased as the infection progressed from 7 to 14 dpi in both transcriptome and proteome levels. Additionally, several GO-enriched categories such as cell wall modification and lignin biosynthetic process were observed in the CS-NILs, where the *P. brassicae* infection was severe. It is known that laccases oxidize phenolic compounds and facilitate the polymerization of lignin and suberin; these hydrophobic polymers play a crucial role in maintaining structural support and rigidity to the cell wall and form a barrier to pathogen invasion and water loss [68]. In this study, we observed a consistently decreased abundance of laccase protein in the CS-NILs. Previously, we found an increase in the abundance of laccase proteins in CR *B. napus* lines [27]. Thus, our studies provide substantial evidence that laccase may play an important role in host resistance against *P. brassicae*.

We also observed a consistently increased abundance of Kelch motif containing proteins at all time points in the CR-NIL roots, and this observation was also supported by the transcriptome analysis [29], indicating that Kelch motif-containing proteins, such as BnaC02T0374800WE, potentially play a role in clubroot resistance. Limited information is available on the role of this type of protein in disease resistance; however, Zhang et al. [69] demonstrated that proteins containing KELCH repeat (motif) regulate the stability of PHENYLALANINE AMMONIA-LYASE (PAL), a vital enzyme for phenylpropanoid biosynthesis. The phenylpropanoid pathway plays a key role in the production of lignin, which is a major component of the cell wall [70]. Taken together, it is evident that in *B. napus*-*P. brassicae* host−pathogen interaction, the host employs diverse proteins that are directly or indirectly involved in cell wall reinforcement and contribute to resistance.

In the integrated dataset, there are several other proteins (therefore genes) that showed significantly increased expression in the CR-NIL roots, and these genes were related to antioxidant responses, such as *GLUCOSE-6-PHOSPHATE DEHYDROGENASE* (*G6PDH*), *GLUTATHIONE S-TRANSFERASE* (*GST*), *CATALASES*, and *4-HYDROXYPHENYLPYRUVATE DIOXYGENASE* (*4-HPPD*). G6PDH is an important enzyme in the oxidative pentose phosphate pathway (OPPP), which is involved in the production of NADPH [71]. The reductant NADPH is essential for ROS signaling and maintaining the cellular redox state by generating reduced glutathione. This antioxidant defense system plays a crucial role in safeguarding cell wall components from oxidative damage [72,73]. Similarly, CATALASE breaks down hydrogen peroxide into oxygen and water and plays a key role in ROS scavenging and maintaining cellular homeostasis [74]. Catalase activity reduces the uncontrolled accumulation of ROS, thereby indirectly influencing the cell wall-modifying enzymes and protecting the integrity of the cell wall [75,76,77]. The finding from this study on CATALASE response to host−pathogen interaction aligns with the results of our earlier study [27], where an increased abundance of CATALASE-related protein was observed in clubroot-resistant *B. napus* lines in response to *P. brassicae*. Thus, *CATALASE* potentially plays a dual role in both signaling and defense responses that involve maintaining cellular integrity and resilience against biotic stress [78,79]. Another protein 4-HPPD, as we found highly abundant in this study, plays a vital role in the biosynthesis of redox cofactors plastoquinone and tocopherols. These antioxidants reduce oxidative stress by scavenging ROS. Finally, our study also revealed several GSTs that are increased in abundance in the CR-NILs in response to the pathogen infection. These proteins are highly induced during stress response and potentially act as important detoxifying compounds involved in the removal of ROS. A higher abundance of these proteins indicates that resistance in *B. napus* against *P. brassicae* is possibly dependent on ROS-mediated signaling and cell wall reinforcement.

## 4. Study Limitations

We conducted this study with four biological replicates per treatment, which is consistent with the current standards in omics research [80,81]. While the study identified several DEGs/DAPs to be involved in clubroot resistance, the ability to capture the very subtle difference between the NILs may still be limited. Another limitation of this study is that the transcriptome data [29] were mapped to *B. napus* cv. Darmor reference genome, whereas the proteome data were mapped to the *B. napus* cv. Westar reference genome. We minimized the discrepancies by performing BLAST 2.16.0 searches and identified the common genes with > 99% nucleotide sequence similarity; however, the use of two different reference genomes may still have introduced minor error. Small-scale genomic differences between the cultivars, such as single-nucleotide polymorphisms, insertions, and deletions, could affect the precision of integrated transcriptome-proteome analysis. Nevertheless, the identification of the key signaling pathways, such as calcium signaling, ROS, and cell wall reinforcement, based on both omic analyses suggests that the major biological conclusions from this study remain valid.

## 5. Conclusions

This study used NILs with contrasting resistance to *P. brassicae* to generate integrated proteomic and transcriptomic insights into clubroot disease resistance in canola. The NIL-based omics data validated the previously reported molecular responses and revealed additional signals, including the involvement of calcium-signaling pathways, ROS, and their potential crosstalk. Additional evidence also pointed to the potential involvement of phytohormones, glucosinolates, and other resistance-related genes. When transcriptome and proteome data were combined, this study identified some of the key genes potentially involved in maintaining cell wall integrity and modulating plant growth and defense mechanisms, including *PECTIN METHYLESTERASE INHIBITOR*, *KELCH MOTIF*, and *LACCASE*. Additionally, genes related to antioxidant responses, such as *G6PDH*, *GST*, *CATALASES*, and *4-HPPD*, were also identified, highlighting the potential role of ROS detoxification in resistance. These findings suggest that resistance in *B. napus* against *P. brassicae* is likely mediated by ROS-mediated signaling and reinforcement of the cell wall. Taken together, this study provides new insights into the molecular basis of clubroot resistance and lays a foundation for functional validation of the genes to elucidate their roles in host−pathogen interaction. This knowledge can ultimately be used in the molecular breeding of *B. napus* canola for clubroot resistance.

## 6. Materials and Methods

### 6.1. Plant Materials

A set of near-isogenic lines (NILs) contrasting in their resistance to *P. brassicae* pathotype 3H was used in this study. The NILs were developed by crossing a clubroot-resistant *B. napus* line carrying resistance introgressed from *B. rapa* ECD 01 (cv. Debra) [82] and a clubroot-susceptible *B. napus* canola line A04-73NA. The F_1_ was subjected to recurrent backcrossing for four generations (BC_4_) using A04-73NA as the recurrent parent, along with selection for resistance to pathotype 3H in each generation. Clubroot-resistant BC_4_ plants were self-pollinated for two generations to develop homozygous resistant and homozygous susceptible BC_4_F_3_ families. Three resistant and three susceptible BC_4_F_3_ families derived from a single heterozygous BC_4_ plant were used in this study; these families hereafter will be referred to as CR-NILs and CS-NILs, respectively.

### 6.2. Preparation of the Inoculum and Inoculation Technique

Single spore isolates of *P. brassicae* pathotype 3H, preserved at −80 °C in the galls of CS *B. napus* cv. Hi-Q, were used. For preparation of the inoculum, frozen galls (37 g) were thawed, homogenized in 1000 mL of distilled water and filtered through multi-layered cheesecloth (American Fibre and Finishing Inc., Albermarle, NC, USA). The density of the resting spores was adjusted to 1 × 10^7^ spores/mL. The NILs were seeded in 32-cell trays filled with Sunshine Professional Growing Mix (Sunshine Horticulture, 15831, N.E., Bellevue, WA, USA), and the plants were grown in a greenhouse at 20–22/15 °C (day/night) temperature, with a 16/8 h (h) photoperiod and a light intensity of 400 µmol/m^2^. For inoculation, 1 mL of the spore suspension was applied to the soil close to the root of the seedlings 10 days after germination. Control plants were treated with sterile water and were maintained in separate trays under the same greenhouse conditions. After inoculation, the soil was kept well saturated with water for about two weeks for successful infection. The plants were fertilized using Nitrogen-Phosphorus-Potassium (20-20-20) once a week.

### 6.3. Sample Collection for Protein Extraction

Based on our earlier study, root infection was observed 7 days post-inoculation [83]. To capture the distinct stages of pathogenesis [83], root samples of both CR-NILs and CS-NILs were collected at three-time points, 7, 14, and 21 days post-inoculation (dpi), and the uninoculated samples were also collected at the same time points. Each root sample included a bulk of three NIL families and seven plants of each NIL family, i.e., a total of 21 plants. Bulking of the NIL families (resistant or susceptible) was performed to minimize any genetic background differences of the NILs. Thus, the experiment included two genotypes (CR-NILs and CS-NILs) and two treatments (pathogen inoculated and uninoculated), and the samples were collected at three time points (7, 14, and 21 dpi); the experiment was replicated four times. Thus, the total number of samples used in this study was 48 (2 genotypes × 3 time points × 2 treatments × 4 biological replicates), and this included growing of 1008 plants (48 × 21).

### 6.4. Protein Extraction for Proteome Analysis

Frozen root tissue samples were ground in liquid nitrogen using a mortar and pestle and were aliquoted for protein extraction. Protein was extracted from 400 mg of ground tissue with a solution of 50 mM HEPES-KOH (pH 8.0), 50 mM NaCl, and 4% (*w*/*v*) SDS at a 1:2 (*w*/*v*) ratio. The protein extract was reduced with 10 mM dithiothreitol (DTT) and alkylated with 30 mM iodoacetamide (IA). Peptide pools were generated using a KingFisher APEX (Thermo Scientific, Waltham, MA, USA) automated sample preparation device as outlined by Leutert et al. [84] without deviation. Samples were digested with sequencing grade trypsin (V5113; Promega, Madison, WI, USA) and acidified with formic acid to a final concentration of 5% (*v*/*v*). Following digestion, samples were acidified with formic acid (A117, Fisher) to a final concentration of 0.5% (*v*/*v*). Peptides were desalted as previously described [85] using an OT-2 liquid handling robot (Opentrons Labworks Inc., Brooklyn, NY, USA) mounted with Omix C18 pipette tips (A5700310K; Agilent, Santa Clara, CA, USA). Desalted peptides were dried and stored at −80 °C prior to re-suspension in 3.0% (*v*/*v*) ACN/0.1% (*v*/*v*) FA and before MS injection.

### 6.5. Nanoflow LC-MS/MS Analysis

Peptides were analyzed using a Fusion Lumos Tribrid Orbitrap mass spectrometer (Thermo Scientific) operated in the data-independent acquisition (DIA) mode, using the previously described BoxCarDIA method [86]. One microgram of re-suspended peptide was injected using an Easy-nLC 1200 system (LC140; ThermoScientific) and a 25 cm Easy-Spray PepMap C18 Column (ES902; Thermo- Scientific). A 40 min nonlinear gradient was used as described previously [86] with peptide eluted with a solvent B gradient (0.1% (*v*/*v*) FA in 80% (*v*/*v*) ACN) consisting of 4–41% B. BoxCarDIA MS1 analysis was performed by using two multiplexed targeted SIM scans of 10 BoxCar windows each as previously described [86]. Detection was performed at a 120,000 resolution and normalized AGC targets of 100% per BoxCar isolation window. MS2 analysis was performed at a resolution of 30,000 using twenty-eight 38.5 *m*/*z* windows with an overlap of 1 *m*/*z* and an AGC target value of 2000%.

### 6.6. Functional Annotation and Enrichment of Differentially Abundant Proteins

All acquired raw files were processed with Spectronaut v17 (Biognosys AG) using default settings without N-acetyl variable modification. Spectra were searched using the published *B. napus* cv. Westar proteome [87]. Significantly changing DAPs were determined and corrected for multiple comparisons (Bonferroni-corrected *p*-value < 0.05; *q*-value). To gain a better understanding of the biological roles, the predicted proteins were annotated with gene descriptions from Kyoto Encyclopedia of Genes and Genomes (KEGG; https://www.genome.jp/kegg/; date accessed: 30 May 2023) [88], Pfam [89], and SwissProt (https://www.expasy.org/resources/uniprotkb-swiss-prot; date accessed: 30 May 2023) databases. The TAIR (https://www.arabidopsis.org; date accessed: 30 May 2023) hit IDs corresponding to the significant *B. napus* proteins (*q*-value < 0.05) were used for gene ontology analysis using the PANTHER Overrepresentation Test in the Gene Ontology database (http://geneontology.org/docs/go-enrichment-analysis/; date accessed: 30 May 2023). The parameters used for this analysis were statistical test type—Fisher’s exact test, correction method—FDR (False Discovery Rate), and a significance level of 0.05. Finally, the TAIR IDs were used for the KEGG pathway analysis using the Database for Annotation, Visualization and Integrated Discovery (DAVID) [90]. The KEGG-enriched categories with an FDR-corrected *p*-value of <0.05 were considered for further analysis after Benjamini−Hochberg correction was applied to multiple testing.

### 6.7. Integration of Transcriptomics with Proteomics

To gain a better understanding of molecular changes that occur during interactions between *B. napus* and *P. brassicae* and to identify the putative resistance genes associated with clubroot resistance, we have integrated transcriptome data of the same CR- and CS-NILs at 7 and 14 dpi [29] with the proteomic data from this study. Differentially expressed genes (DEGs) (*q*-value < 0.05) were selected from RNA-seq data [29]. The RNA-seq analysis was based on the *B. napus* cv. Darmor reference genome, while proteome analysis was based on the *B. napus* cv. Westar reference genomes. We used the Basic Local Algorithm Search Tool (BLAST) to identify the common genes in both transcriptome and proteome datasets [91]. We applied BLASTn command in the Galaxy Server (v24.1.1) to perform nucleotide-level comparisons [92]. Genes exhibiting a 99% sequence similarity at the nucleotide level between Westar and Darmor reference genomes were selected for further analysis. Log_2_ fold-change values for all DEGs and DAPs that were common in both studies were extracted from both datasets. These values were used to analyze gene expression patterns across various time points using R-statistical software (v3.6.3). Furthermore, for all the common *B. napus* gene IDs identified in both studies, the corresponding TAIR IDs were retrieved via the Arabidopsis Information Resource (https://www.arabidopsis.org; date accessed 20 March 2024). These TAIR IDs were subsequently used for gene ontology (GO) analysis using the Database for Annotation, Visualization and Integrated Discovery (DAVID) [90].

### 6.8. Correlation Analysis

To assess the correlation between transcript and protein changes, we focused on genes that are commonly detected in both datasets and are differentially abundant in two or more time points. We applied R statistics to calculate Pearson and Spearman correlation coefficients, and the results were visualized with scatter plots to assess consistency between transcript levels and protein abundance. Furthermore, to check sample quality, we looked at the consistency of the biological replicates by running principal component analysis across time points, treatments, and genotypes, and by calculating pairwise Pearson correlations.

## Data Availability

The data that support the findings of this study are openly available in the PRoteomics IDEntifications Database (PRIDE at https://www.ebi.ac.uk/pride/, reference number: PXD060262 (reviewer credentials username: reviewer_pxd060262@ebi.ac.uk; password: 3VOx1pYHD4Rh)). Further, Supplementary Data associated with this article can be found in the online version of this manuscript.

## Supplementary Materials

The following supporting information can be downloaded at: https://www.mdpi.com/article/10.3390/ijms26189157/s1.

## Abbreviations

CAN—acetonitrile; AEE4—acyl-activating enzyme 4; AGC—automatic gain control; AOP1— alkenyl hydroxyalkyl producing 1; ARF—auxin response factors; AuxRe—auxin-responsive elements; BLAST—basic local algorithm search tool; BP—biological process; CAB—chlorophyll a and b; CAT 2—catalase 2; CC—cellular component; CH25—chitin 25; CHB4—chitinase basic 4; CK—cytokinin; COMT1—caffeic acid 3-O-methyltransferase; CR—clubroot-resistant; CRRSP9—cysteine-rich secretory protein 9; CS—clubroot-susceptible; CSE—caffeoyl shikimate esterase; DAPs—differentially abundant proteins; DAVID—Database for Annotation, Visualization and Integrated Discovery; DEG—differentially expressed gene; DIA—data-independent acquisition; dpi—day post-inoculation; DTT—dithiothreitol; ECI3—enoyl-coA delta isomerase 3; EDR2L—enhanced disease resistance 2-like; ERD10—early response to dehydration 10; ERF—ethylene response factor; ESP—epithiospecifier proteins; ETI—effector-triggered immunity; EXPA15—expansin-A15; FAAH—fatty acid amide hydrolase; FDR—false discovery rate; FER—Feronia; FLS2—flagellin sensitive 2; G6PDH—glucose-6-phosphate dehydrogenase; GAUT4—glucuronosyltransferase 4; GEM—gtp-binding protein overexpressed in skeletal muscle; GO—gene ontology; GST—glutathione s-transferase; GSTU12—glutathione-S-transferase U12; GULLO2—gluconolactone oxidase 2; HEPES-KOH—4-(2-hydroxyethyl)-1-piperazineethanesulphonic acid; 4-HPPD—4-hydroxyphenylpyruvate dioxygenase; HST—hydroxycinnamoyl transferase; IA—iodoacetamide; IAA—indole-3-acetic acid; ILL2—IAA ILR1-like; ILR1—IAA leucine-resistant 1; IQD—isoleucine glutamine domain; JA—jasmonic acid; KEGG—Kyoto Encyclopedia of Genes and Genomes; KOR2—korrigan; LRR-RLK—leucine-rich repeat receptor-like kinase; MAPK—mitogen-activated protein kinase; MATH—meprin and TRAF homology; MF—molecular function; MLO—mildew locus O; MLP—major latex protein-like protein; mRNA—messenger ribonucleic acid; MS—mass spectrometry; NaCl—sodium chloride; NILs—near-isogenic lines; NSP—nitrile-specifier protein; NSP 2—nitrile specifier protein 2; OPPP—oxidative pentose phosphate pathway; OPR1—12-oxophytodienoate reductase 1; PAL—phenylalanine ammonia-lyase; PAMP—pathogen-associated molecular pattern; Pathotype 3H—pathotype group (3) and new CCD system (letter H); PED1—peroxisome deficient 1; Per—peroxidase; PIN—pin formed; PR1—pathogenesis-related 1; PRXC2—peroxidase C2; PTI—PAMP-triggered immunity; PXG3—peroxygenase 3; *q*-value—quantile value; RAF—rapid alkalinization factor; RALF—rapid alkalinization factor; RC—resistant control; Rcr1—resistance to clubroot 1; RF2b—repressor factor 2b; RGLG2—ring domain ligase 2; RI—resistant inoculated; RIN4—rpmi1-interacting protein 4; ROS—reactive oxygen species; RPMI1—resistance to Pseudomonas syringae pv. Maculicola 1; RPN1B—regulatory particle non-ATPase 1b; SA—salicylic acid; SAG12—senescence-associated gene 12; SC—susceptible control; SDR1—suppressor of disease resistance 1; SDS—sodium dodecyl sulfate; SI—susceptible inoculated; SR—splicing regulator; TAIR—the Arabidopsis information resources; TGA6—tgacg motif-binding factor 6; TIP1—tonoplast intrinsic protein 1; TPS—tricyclene synthase; TPS10—terpene synthase 10; TRX5—thioredoxin H5; UBP12—ubiquitin-specific protease 12; UDP—uridine diphosphate; XTH22—xyloglucan endotransglucosylase/hydrolase protein 22; XXT2—xyloglucan-6-xylosyltransferase 2; YSL9—yellow leaf-specific protein 9.
