# Supplementary material for: Proteomics Integrated with Transcriptomics of Clubroot Resistant and Susceptible Brassica napus in Response to Plasmodiophora brassicae Infection
Source: Int J Mol Sci. 2025 Sep 19;26(18):9157. doi: 10.3390/ijms26189157 (PMC12470197; doi:10.3390/ijms26189157)
Supplement: Supplementary file 1 [file ijms-26-09157-s001.zip › Figure S1.pdf]

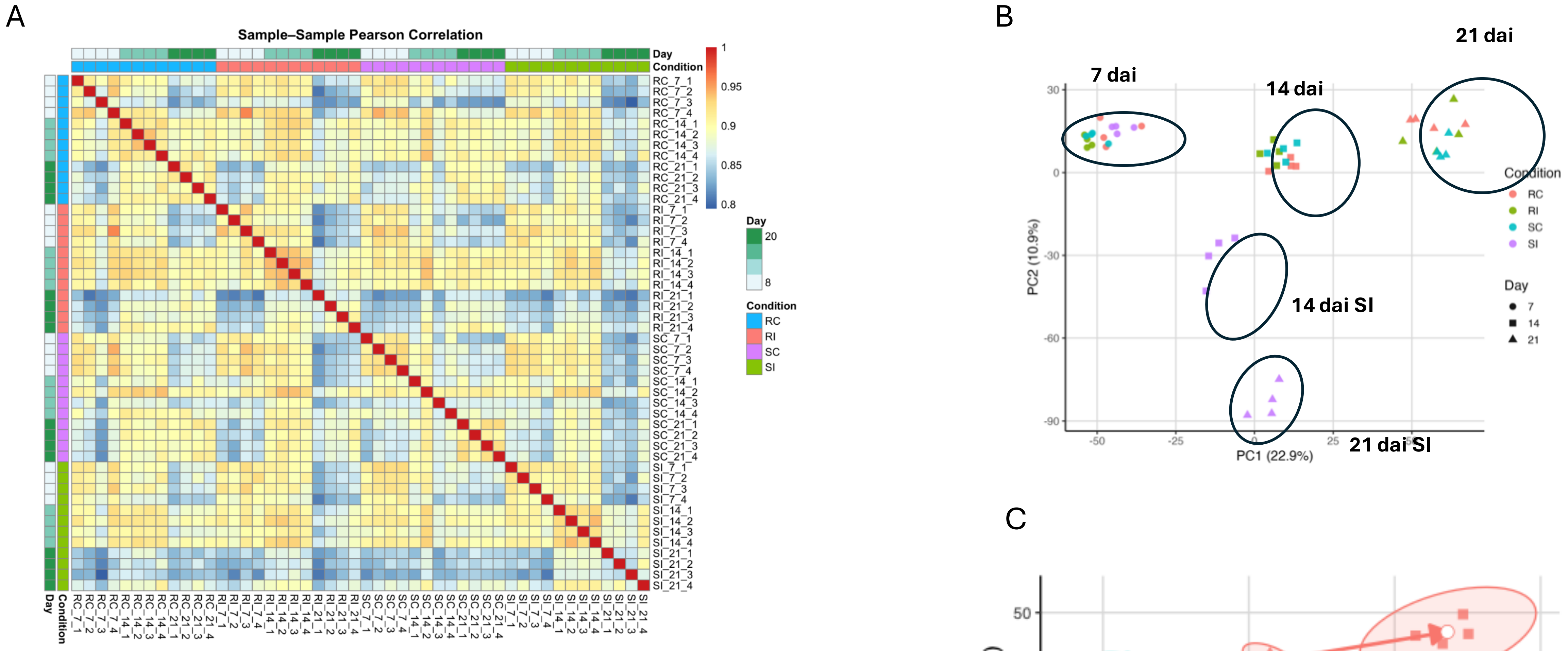


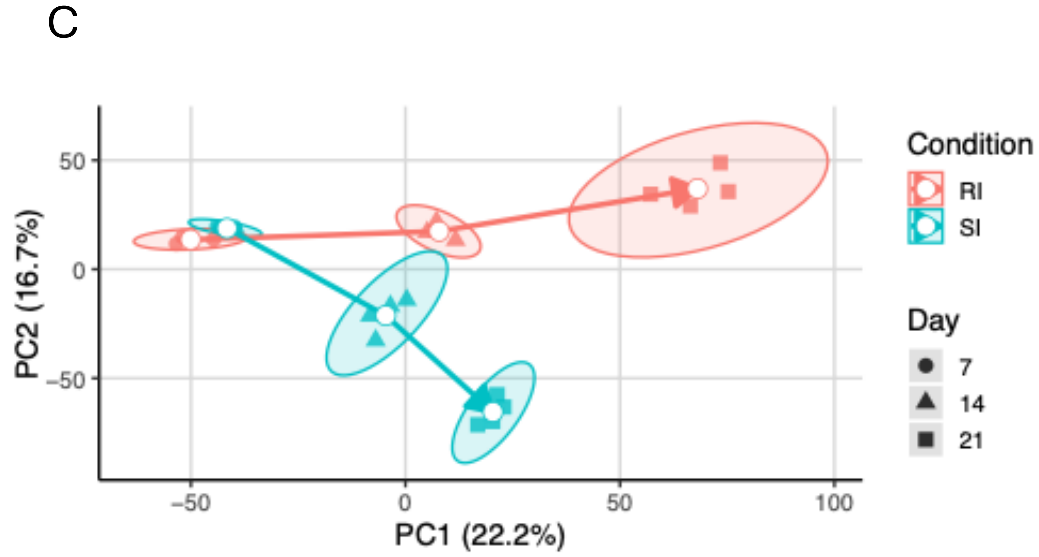


**Figure** S1. Proteome data quality assessment. (A) Sample to sample Pearson correlation. (B) Principal component analysis (PCA) of biological replicates across all time points and genotypes. (C) PCA of biological replicates within inoculated resistant and inoculated samples with 95% confidence ellipses and time points.
