# Supplementary figures and images for: Proteomics Integrated with Transcriptomics of Clubroot Resistant and Susceptible Brassica napus in Response to Plasmodiophora brassicae Infection
Source: Int J Mol Sci. 2025 Sep 19;26(18):9157. doi: 10.3390/ijms26189157 (PMC12470197; doi:10.3390/ijms26189157)

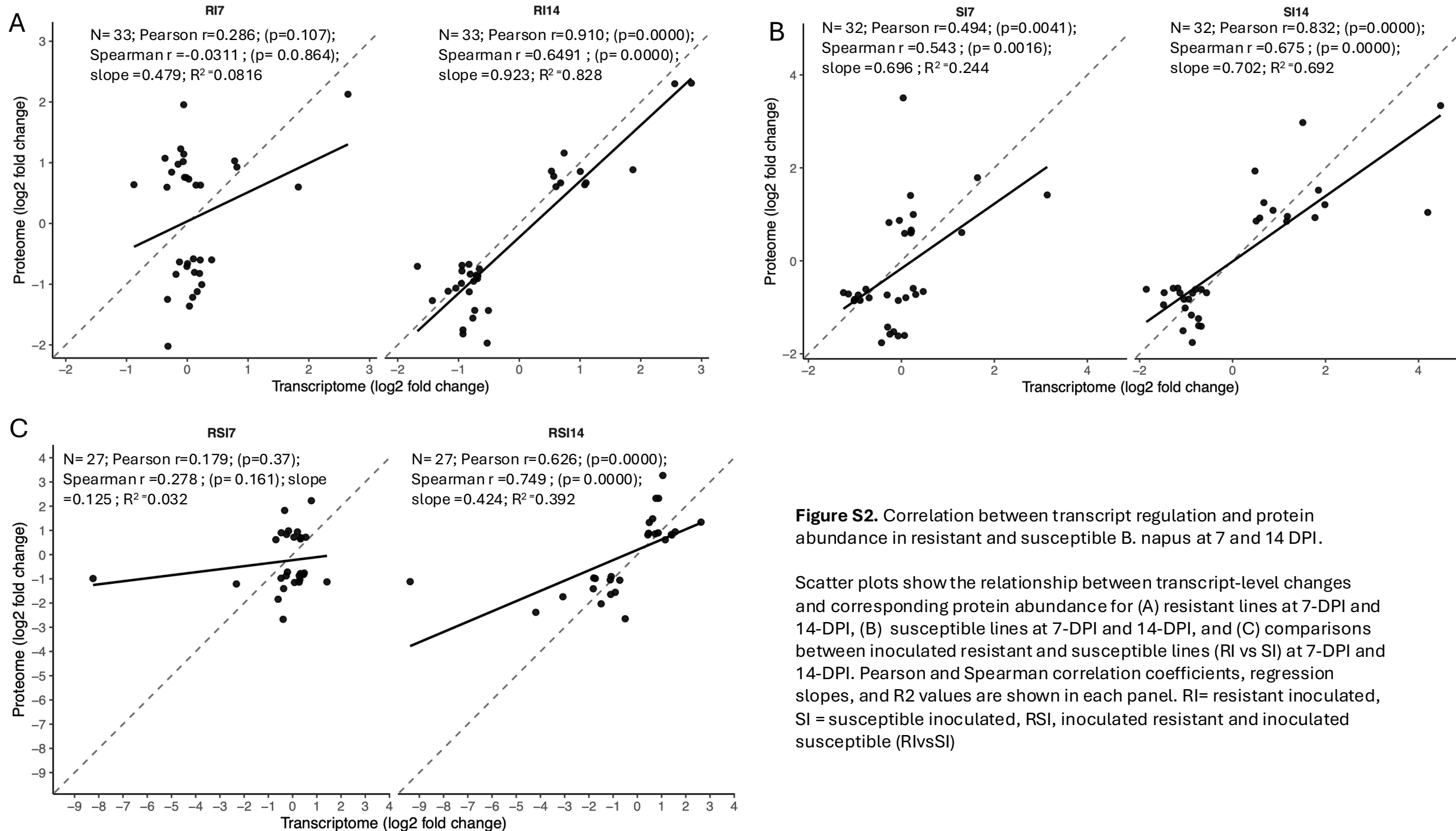

Supplement: Supplementary file 1 [file ijms-26-09157-s001.zip › Figure S2.pdf]
